# Structural Basis of the Binding Mode of the Antineoplastic Compound Motixafortide (BL-8040) in the CXCR4 Chemokine Receptor

**DOI:** 10.3390/ijms24054393

**Published:** 2023-02-23

**Authors:** Mariana Rebolledo-Bustillo, David Garcia-Gomez, Eliud Morales Dávila, María Eugenia Castro, Norma A. Caballero, Francisco J. Melendez, Victor M. Baizabal-Aguirre, Brenda L. Sanchez-Gaytan, Jose Manuel Perez-Aguilar

**Affiliations:** 1 School of Chemical Sciences, Meritorious Autonomous University of Puebla (BUAP), University City, Puebla 72570, Mexico; 2 Chemistry Center, Science Institute, Meritorious Autonomous University of Puebla (BUAP), University City, Puebla 72570, Mexico; 3 School of Biological Sciences, Meritorious Autonomous University of Puebla (BUAP), University City, Puebla 72570, Mexico; 4 Centro Multidisciplinario de Estudios en Biotecnología, Facultad de Medicina Veterinaria y Zootecnia, Universidad Michoacana de San Nicolás de Hidalgo, Km. 9.5 s/n Carretera Morelia-Zinapécuaro, La Palma, Tarímbaro, Morelia 58893, MICH, Mexico

**Keywords:** cancer, computational methods, MD simulations, motixafortide, antineoplastic drugs, CXCR4

## Abstract

Modulation of the CXCL12–CXCR4 signaling axis is of the utmost importance due to its central involvement in several pathological disorders, including inflammatory diseases and cancer. Among the different currently available drugs that inhibit CXCR4 activation, motixafortide—a best-in-class antagonist of this GPCR receptor—has exhibited promising results in preclinical studies of pancreatic, breast, and lung cancers. However, detailed information on the interaction mechanism of motixafortide is still lacking. Here, we characterize the motixafortide/CXCR4 and CXCL12/CXCR4 protein complexes by using computational techniques including unbiased all-atom molecular dynamics simulations. Our microsecond-long simulations of the protein systems indicate that the agonist triggers changes associated with active-like GPCR conformations, while the antagonist favors inactive conformations of CXCR4. Detailed ligand–protein analysis indicates the importance of motixafortide’s six cationic residues, all of which established charge–charge interactions with acidic CXCR4 residues. Furthermore, two synthetic bulky chemical moieties of motixafortide work in tandem to restrict the conformations of important residues associated with CXCR4 activation. Our results not only elucidate the molecular mechanism by which motixafortide interacts with the CXCR4 receptor and stabilizes its inactive states, but also provide essential information to rationally design CXCR4 inhibitors that preserve the outstanding pharmacological features of motixafortide.

## 1. Introduction

Cancer is a collection of diseases that has become a central problem and a priority in public health, since it represents the leading cause of mortality and morbidity worldwide [1]. Described as a pathogenic process of uncontrolled growth and dissemination of cells, resulting in a disease with the ability to infiltrate, destroy normal body tissue, and spread throughout the body, cancer caused approximately 10 million deaths and 19.3 million new cases in 2020 [2].

Due to the complexity of cancer, several approaches are needed to either prevent or reduce its progression. In this regard, the incorporation of different techniques to tackle most of cancer’s deleterious features—including those that can provide information at the molecular level—is essential. Computational techniques have been applied to understand protein function and may provide reliable information about the ligand-binding mechanisms of pharmacologically relevant molecules [3,4]. Detailed information about the interacting determinants of the ligand–protein complex is fundamental not only to mitigate the complex process of drug resistance that commonly arises in antineoplastic drugs, but also to design chemical modifications to circumvent such resistance. in advance.

Approaches that combine typical chemotherapy with new drugs to provide a synergistic treatment have been introduced in recent years. One particularly interesting anticancer drug candidate is the peptide motixafortide (BL-8040). This best-in-class antagonist of the CXCR4 receptor was developed by *BiolineRx* and has been utilized in preclinical studies on pancreatic, breast, and lung cancers (commonly administered subcutaneously), with encouraging results in terms of the observed augmented benefits of chemotherapy [5]. Nonetheless, molecular-level information about the interaction mechanism of motixafortide at the human CXCR4 is lacking.

To contribute to the understanding of the pharmacological activity of motixafortide in inhibiting the CXCL12–CXCR4 signaling axis, we utilized well-established computational methods to characterize the binding mode of the motixafortide/CXCR4 complex. Using unbiased all-atom molecular dynamics simulations, we described the main interactions involving the antagonist as well as the conformational consequences in the receptor. Our results of the interacting determinants of the inhibitor are discussed in the context of microsecond-long simulations of the protein complex formed by the CXCR4 receptor and the CXCL12 chemokine (CXCL12 is the endogenous ligand of the CXCR4 receptor). Starting from the same initial structure of the CXCR4 receptor, we found that the computational methods utilized here were able to distinguish the conformational changes elicited by either ligand—the agonist (CXCL12) or the antagonist (motixafortide). That is, in the presence of the CXCL12, different structural motifs in CXCR4 adopt conformations associated with active-like (i.e., agonist-bound) GPCR structures. In contrast, in the presence of motixafortide, inactive conformations of CXCR4 were found. Next, the interacting determinants of both ligands were analyzed, showing not only that the cationic character of motixafortide is important to explain its high affinity (IC_50_ of 0.54–4.5 nM) and long occupancy (>48 h), but also that the presence of the unnatural chemical moieties borne in its structure exhibits notable relevance.

Our results provide molecular-level information regarding the binding mode of motixafortide that explains its outstanding antineoplastic properties, while also dissecting the contribution of its unique constituent amino acid sequence. This information is critical to design derivatives of this compound that retain its outstanding anticancer properties.

## 2. Results

**Figure 1:**
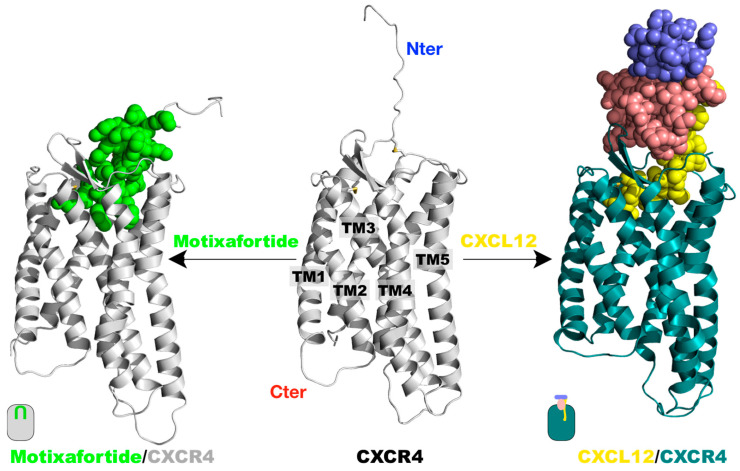
Protein complexes investigated: The tertiary structure of the CXCR4 receptor was taken from the X-ray structure with the PDB accession code 4RWS.pdb. A segment at the N-terminus was modelled as an extended structure (central panel). The same CXCR4 structure was utilized to generate the two protein complexes with either CXCL12 or motixafortide, by placing the ligands at the CXCR4 orthosteric binding site as described in the Materials and Methods section. The systems were embedded in a hydrated lipid bilayer and investigated by 1.0-microsecond-long unbiased MD simulations. A representative final structure of the motixafortide/CXCR4 complex is depicted in the left panel, where the CXCR4 structure is colored grey, while motixafortide is depicted as green spheres. In the right panel, an equivalent final structure of the CXCL12/CXCR4 complex is shown, where the CXCR4 receptor is colored teal, while the chemokine is presented as spheres using the same color code as in Figure S1.

To investigate the interacting determinants of motixafortide, we constructed its complex with the human CXCR4 receptor (Figure 1 and Figure S1). Additionally, and to contextualize the results, the protein complex formed by the CXCR4 receptor with its endogenous ligand—the stromal cell-derived factor 1 (SDF-l or CXCL12)—was also generated (Figure 1 and Figure S1); details about the generation of the two protein complexes can be found in the Materials and Methods section. Both motixafortide/CXCR4 and CXCL12/CXCR4 were embedded in a hydrated phospholipid bilayer and investigated by extensive unbiased molecular dynamics (MD) simulations (Figure S2).

To evaluate the capacity of our computational characterization to capture the detailed structural elements associated with GPCR activation, the receptor’s conformational consequences triggered by the presence of each ligand were investigated. Several conserved structural motifs have been associated with GPCR activation, including the PIF and NPxxY motifs, as well as the toggle switch at TM6 (W^6.48^). Since both all-atom MD simulations started from the same CXCR4 structure, different conformations adopted by the receptor should be attributed to the presence of either an agonist ligand (CXCL12) or an antagonist ligand (motixafortide) (see also Figure 1 and Figure S3).

**Figure 2:**
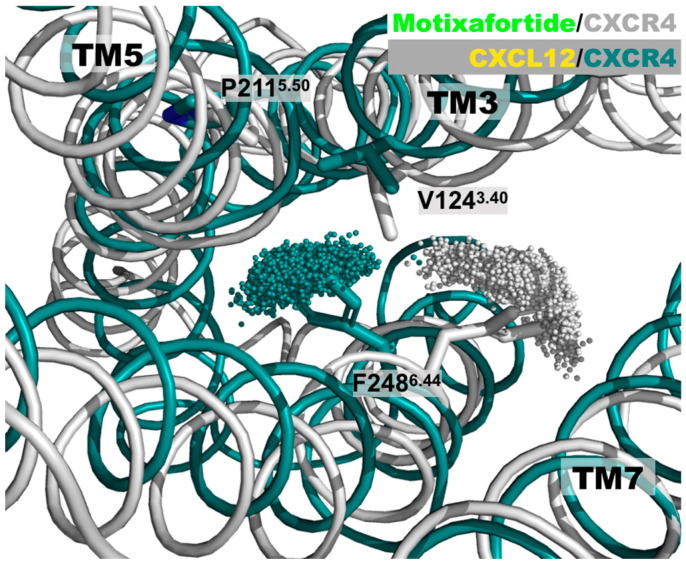
Distinct CXCR4 conformations of the PIF motif: A superimposed representation of the positions adopted by the F248^6.44^ aromatic residue. For the sake of clarity, the conformations adopted by the sidechain of the F248^6.44^ residue during the last 200 ns are represented by the position of the most external carbon in the phenyl ring—that is, the ‘CZ’ atom. The CXCR4 structure in the motixafortide/CXCR4 complex is depicted in grey, while that of the CXCL12/CXCR4 complex is depicted in teal. Even though the initial structure of the CXCR4 receptor in both protein complexes is the same, the presence of either the agonist (CXCL12) or the antagonist (motixafortide) ligand elicits distinct conformations on this important structural motif, associated with GPCR activation. These conformations are consistent with the expected rotameric states adopted by the F248^6.44^ residue (see also Figure S4).

*The PIF motif*: In several prototypical class A GPCRs, the conserved PIF motif is constituted by the P^5.50^, I^3.40^, and F^6.44^ hydrophobic residues (the superscripts display the Ballesteros–Weinstein numbering). In the case of the human CXCR4 receptor and various class A receptors, a valine replaces the isoleucine residue at position 3.40; that is, the triad of hydrophobic residues that constitute this important motif is formed by P211^5.50^, V124^3.40^, and F248^6.44^. Upon receptor activation induced by the presence of an agonist ligand, there is a repacking of this motif that contributes to the distinctive outward movement of the intracellular segment of TM6 (and the less dramatic inward movement of TM7) [6,7]. As observed in the active and inactive structures of a prototypical class A GPCR—the β2 adrenergic receptor—the residue at position 6.44 (F^6.44^) exhibits a significant change (Figure S4). That is, relative to the inactive conformation, residue F^6.44^ moves toward TM5 in the active state. Analysis of the conformations adopted by the F248^6.44^ residue in both CXCL12/CXCR4 and motixafortide/CXCR4 complexes indicated different behavior in the residue sidechain (Figure 2). During the simulation, a change in the orientation of the F248^6.44^ residue toward TM5 occurred only in the agonist-bound system, while in the antagonist-bound system the bulky sidechain librated around the initial position, corresponding to the inactive CXCR4 state (4RWS.pdb). Notably, the conformations adopted by the F248^6.44^ residue in the CXCL12/CXCR4 system were associated with active structures of the receptor, while those observed in the motixafortide/CXCR4 system were linked to inactive conformations of the receptor (see also Figure S4) [8].

**Figure 3:**
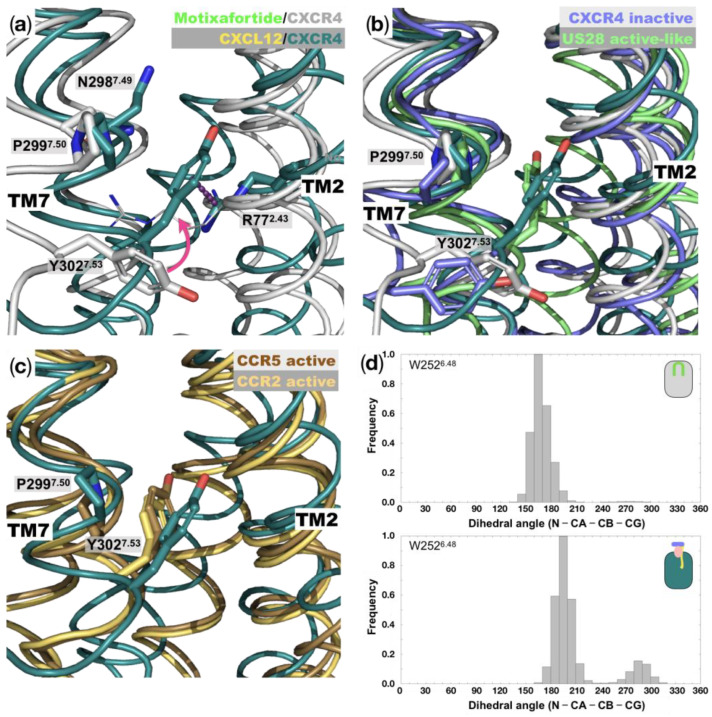
Conformations at the NPxxY and toggle switch motifs: (**a**) Representative structures of the final stages of the MD simulation for the motixafortide/CXCR4 and CXCL12/CXCR4 protein complexes. Residues that are part of the highly conserved NPxxY motif, which has been implicated in GPCR activation, are indicated (N298^7.49^, P299^7.50^, and Y302^7.53^); TM6 is not depicted in the figure. The position of residue Y302^7.53^ changes significantly in the agonist-bound CXCR4 complex (colored teal), while in the antagonist-bound system (grey) it remains very similar to the starting conformation (see magenta arrow). (**b**) Comparison of the NPxxY motifs of the two CXCR4 complexes investigated here with the inactive structure of CXCR4 (4RWS.pdb) and the active-like structure of the viral chemokine receptor US28 (4XT3.pdb). While the sidechain position of residue Y302^7.53^ in the motixafortide/CXCR4 complex resembles that of the inactive CXCR4 structure, the sidechain in CXCL12/CXCR4 moves towards the interior of the helical bundles, as in the case of the active-like GPCR structure. (**c**) Superposition of the agonist-bound system (CXCL12/CXCR4) with the active structures of the closely related CCR5 (7F1S.pdb) and CCR2 (7XA3.pdb) chemokine receptors, showing the resemblance in the conformation of Y302^7.53^ from the highly conserved NPxxY motif. (**d**) The dihedral angle defined by the N-CA-CB-CG atoms from the *toggle switch* residue, W252^6.48^, are shown for the antagonist-bound and agonist-bound CXCR4 systems.

*The NPxxY motif*: The NPxxY structural motif is highly conserved in class A GPCRs and plays a significant role in GPCR activation. Particularly, it has been proposed that upon activation, the aromatic residue Y302^7.53^ swaps interhelical interactions that facilitate the outward movement of the intracellular segment of TM6, which is a hallmark of GPCR activation [6,7]. In this regard, analysis of our simulation results indicates that the Y302^7.53^ residue displays different conformations in the two CXCR4 complexes. In motixafortide/CXCR4, residue Y302^7.53^ points its bulky sidechain towards the intracellular side, forming polar interactions with residue D74^2.40^. In contrast, in the CXCL12/CXCR4 system, the sidechain points toward the center of the helical bundle—particularly toward TM3 (Figure 3a). The conformation of Y302^7.53^ in the agonist-bound system is stabilized by a cation–pi interaction with residue R77^2.43^ (Figure 3a). As shown in Figure 3b, when we compare the simulation results regarding the Y302^7.53^ conformations with those observed in the inactive CXCR4 structure (4RWS.pdb) and the active-like US28 structure (4XT3.pdb)—a viral chemokine receptor—it shows the similarity of the Y302^7.53^ conformation from those experimentally solved structures with the antagonist- and agonist-bound receptor conformations, respectively (Figure 3b). That is, the conformations adopted by Y302^7.53^ in the motixafortide/CXCR4 system mirror those observed in the inactive X-ray structure of the receptor, while those observed in the CXCL12/CXCR4 system resemble the conformation adopted in the active-like receptor structure. Lastly, a comparison of the results from the CXCL12/CXCR4 system with the active structures of the closely related chemokine receptors CCR5 (7F1S.pdb) and CCR2 (7XA3.pdb) indicates a similarity in the positions of the Y302^7.53^ residue (Figure 3c).

*The toggle switch motif (W^6.48^)*: As mentioned previously, a hallmark in GPCR activation is the conformational change involving the concerted outward and rotational movement of TM6 that results in a separation of the intracellular segment of TM6 relative to the helical bundle by approximately 10 Å [7,8]. Such conformational change is required for the coupling of the respective G protein and is initiated by an extracellular stimulus, which in the case of the CXCR4 receptor is constituted by the binding of the CXCL12 chemokine. The conformational changes that occur at the intracellular side of the receptor are strongly coupled with changes in the rotameric states of residue W^6.48^. This bulky residue located in TM6 is known as the *toggle switch*, and it has been strongly linked to GPCR activation [6,7]. This residue, together with residue P^6.50^, forms part of the highly conserved CWxP motif. Analysis of the rotameric states of residue W252^6.48^—particularly the dihedral angle formed by the N-CA-CB-CG atoms—indicates a different behavior between the agonist- and antagonist-bound systems. As shown in Figure 3d, the values observed in the CXCL12/CXCR4 complex present a bimodal distribution that is more diverse than the motixafortide counterpart—conformational changes in the TM6 *toggle switch* are associated with the presence of an agonist ligand.

Since the computational methods were able to identify the detailed conformational differences associated with agonist-bound and antagonist-bound CXCR4 systems, we characterized the interactions of motixafortide in the orthosteric binding site of the human CXCR4 receptor at the molecular level. As indicated by the calculation of the RMSD, the molecular pose of motixafortide in the orthosteric binding site of CXCR4 remains very stable (see Figure S5 and Video S1). Moreover, the secondary structure of motixafortide is preserved along the entire trajectory; that is, motixafortide retains its β-sheet secondary structural character throughout the MD simulation (Figure S6).

**Figure 4:**
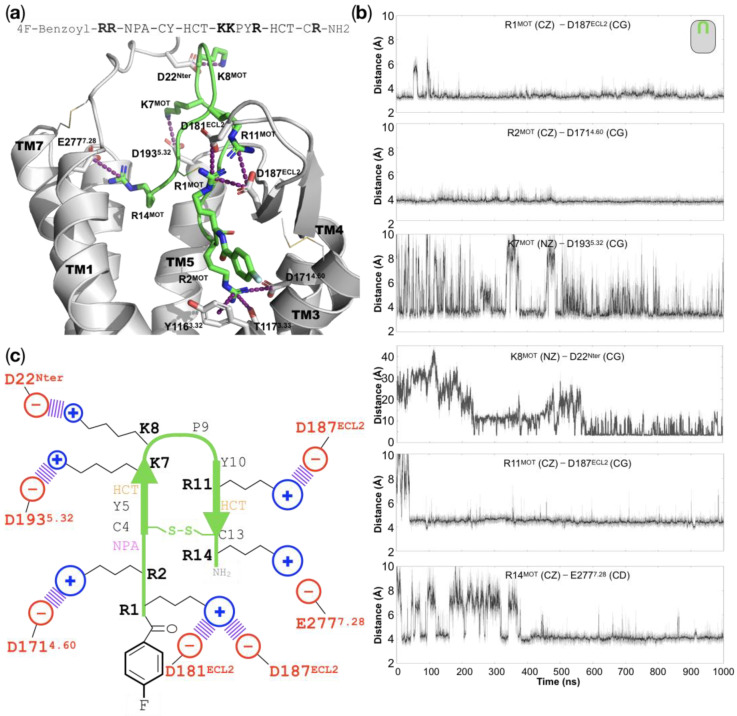
Interactions in the motixafortide/CXCR4 protein complex: (**a**) Representative structure of the final stages from the atomistic MD simulation of the motixafortide/CXCR4 complex, where we can observe that all of the cationic residues of motixafortide are involved in charge–charge interactions with residues in the ligand-binding site of CXCR4 (see purple dashed lines). For the sake of clarity, TM6 is not displayed, and the sequence of motixafortide is depicted at the top of the panel, where its six basic residues (R1, R2, K7, K8, R11, and R14) are highlighted. (**b**) The time evolution plots of distances involving the interactions in the six basic residues from motixafortide (the moving average is colored black, while the primary data are colored gray). (**c**) Schematic representation of the charge–charge interactions that stabilize the molecular pose of motixafortide in the orthosteric binding site of CXCR4 (see also Video S1).

*All of the basic residues in motixafortide establish charge–charge interactions with residues in the CXCR4 orthosteric binding site*: The 14-residue peptide motixafortide contains six cationic residues, four arginines, and two lysines (R1, R2, K7, K8, R11, and R14; see Figure S7). Throughout our simulations we observed that these basic residues were mainly involved in charge–charge interactions, which seemed to be the driving force behind the high affinity of motixafortide for the CXCR4 receptor (see Video S1). R1^MOT^, which contains an unnatural capping group (see the Discussion section), establishes electrostatic interactions with two acidic residues located at ECL2—D181^ECL2^ and D187^ECL2^, which were conserved throughout the entire simulation (see Figure 4). R2^MOT^ is inserted deeper in the ligand-binding site, where it forms charge–charge interactions with residue D171^4.60^. Additionally, R2^MOT^ forms polar interactions and cation–pi interactions with two residues in TM3: T117^3.33^ and Y116^3.32^, respectively. Interestingly, position Y116^3.32^ has been suggested to play an important role in the receptor’s activation for other GPCR members [9]. The neighboring residues K7^MOT^ and K8^MOT^ both interact with aspartate residues. The former interacts with residue D193^5.32^, which is located at the beginning of TM5, while the latter interaction involves residue D22 at the N-terminal segment of CXCR4, which was not present in the initial complex structure but was formed after the second half of the simulation and remained relatively stable for the rest of the trajectory. Similarly, the charge–charge interaction between R11^MOT^ and D187^ECL2^ was not present in the initial complex structure but formed relatively quickly and remained very stable throughout the simulation. Lastly, the interaction between R14^MOT^ and E277^7.28^ was formed relatively quickly and, after a formation–disruption phase that lasted around 400 ns, this interaction remained stable for the rest of the duration of our simulation. That all of the negatively charged residues in motixafortide interact with positively charged counterparts in the receptor’s binding site may be the driving force behind its high affinity (IC_50_ of 0.54–4.5 nM) and long occupancy (>48 h) for the CXCR4 receptor (see Figure 4 and Video S1) [10].

*The unnatural chemical moieties in motixafortide establish aromatic interactions at different places in the CXCR4 orthosteric binding site that restrict changes associated with activation*: The structure of motixafortide contains various chemical groups beyond the standard α-amino acids, including an unnatural N-terminal capping group, the α-amino acid citrulline, and a synthetic naphthalene derivative (Figure S7). Through our simulations, we investigated the possible interaction mechanisms and conformational consequences of the presence of these chemical groups.

**Figure 5:**
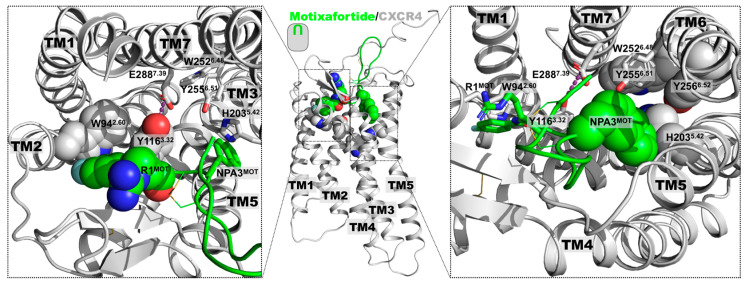
Interactions of the unnatural moieties of motixafortide: The two synthetic components in motixafortide are a 4-fluorobenzoyl group as an N-terminal capping group and a naphthalene derivate (NPA) sidechain at position 3 (see also Figure S7). The results from the unbiased MD simulations placed the two chemical moieties at different locations inside the orthosteric binding site (lateral view of the central panel). As shown in the left panel (extracellular view), the 4-fluorobenzoyl forms aromatic interactions with residues W94^2.60^ and Y116^3.32^. The presence of the 4-fluorobenzoyl group restricts the conformation of this bulky residue, facilitating the interaction of the hydroxyl group of the Y116^3.32^ sidechain with E288^7.39^ (purple dashed line). As shown in the right panel (extracellular view), the NPA sidechain forms aromatic interactions with H203^5.42^, and it is placed in the vicinity of residues in TM6—particularly Y255^6.51^.

**Figure 6:**
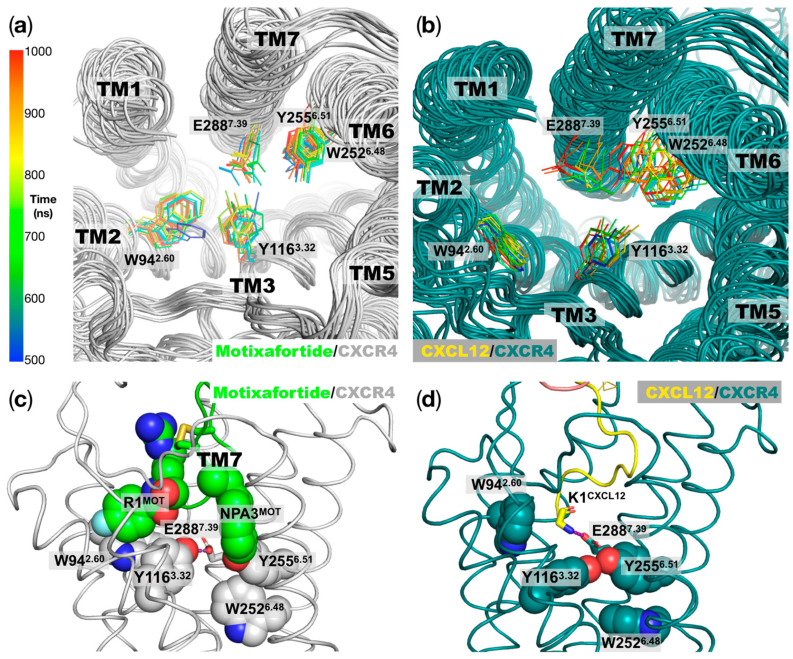
Conformations of bulky residues at the orthosteric binding site linked to CXCR4 activation: (**a**) Motixafortide/CXCR4 superimposed conformations of residues important for ligand binding and activation at the CXCR4 orthosteric ligand binding site. The structures were taken every 50 ns for the last 500 ns of the trajectory (500 to 1000 ns) and are depicted according to the gradient color code on the right panel. (**b**) Similar representation as in panel (**a**), but for the CXCL12/CXCR4 system. In panels (**a**,**b**) we can observe more dynamic behavior in the agonist-bound system relative to the antagonist-bound CXCR4 receptor for particular residues that have been implicated in ligand binding and CXCR4 activation. (**c**) Representative structure of the motixafortide/CXCR4 complex, where the two synthetic chemical moieties at positions 1 and 3 and the CXCR4 residues in their close proximity are depicted as spheres. The polar interaction of residue Y116^3.32^ with E288^7.39^ is highlighted using a purple dashed line. (**d**) Similar orientation as in panel (**c**), but for the CXCL12/CXCR4 protein complex. Here, the polar interaction of residue Y116^3.32^-E288^7.39^ is replaced by the Y116^3.32^-Y255^6.51^ polar interaction (purple dashed line). In this case, the positively charged moiety in the K1^CXCL12^ backbone establishes a charge–charge interaction with E288^7.39^.

*4-Fluorobenzoyl N-terminal capping group*: As shown in Figure S7, there is a synthetic chemical group in residue R1 of motixafortide. Our simulations indicated that the 4-fluorobenzoyl group is positioned in proximity of TM2 and TM3, where it establishes aromatic interactions with residues W94^2.60^ and Y116^3.32^ (Figure 5, left panel). Moreover, this hydrophobic group interacts with C186 in ECL2, which forms a disulfide bond with a cysteine residue at TM3; this disulfide bond is highly conserved in class A GPCRs [11]. Interestingly, the presence of the aromatic ring in the N-terminal capping group of motixafortide seems to stabilize the position of the two bulky residues, W94^2.60^ and Y116^3.32^ (Figure 6a), which remained relatively close to one another throughout the simulation (Figure 5, left panel). In particular, the limited conformational space adopted by the sidechain of Y116^3.32^ positions its hydroxyl group close to position E288^7.39^, where they can establish polar interactions (left panel in Figure 5 and Figure 6b). Notably, position Y116^3.32^ has been proposed to play an important role in CXCR4 activation and CXCL12 binding, and it has been classified as part of a group known as signal initiator residues [11]. In particular, mutagenesis studies of the CXCR4 receptor haven shown that the mutation of position Y116^3.32^ by either a serine or an alanine residue (Y116^3.32^S and Y116^3.32^A, respectively) causes a decrease in the CXCR4 receptor’s activation when stimulated with the endogenous ligand CXCL12 [12,13]. In contrast, in the agonist-bound system, W94^2.60^ and Y116^3.32^ move away from one another because of the absence of an aromatic anchor, as in the case of the of the 4-fluorobenzoyl N-terminal capping group of motixafortide. In this case, the CXCL12 ligand places its N-terminal residue K1^CXCL12^ in this region, where the positively charged backbone amino group establishes charge–charge interactions with E288^7.39^ (Figure 6d). Moreover, the polar sidechain of Y116^3.32^ forms interactions with the sidechain of Y255^6.51^. The latter residue strongly influences the conformations adopted by the highly conserved toggle switch of CXCR4 at TM6 (W252^6.48^). As shown in Figure 6b, in the agonist-bound system, both bulky residues at TM6—Y255^6.51^ and W252^6.48^—display more dynamic behavior relative to the case of the antagonist-bound system (Figure 6b), which can be partially attributed to the presence of the 4-fluorobenzoyl N-terminal capping group of motixafortide.

*A naphthalene derivative sidechain in position 3*: As observed in Figure S7, position 3 of motixafortide bears a synthetic sidechain—namely, NPA (2-naphthyl-alanine). Relative to the position of the 4-fluorobenzoyl group, this bulky aromatic sidechain is placed in the opposite side of the orthosteric binding pocket, in proximity to TM5 (Figure 5, right panel). At this position, the naphthalene derivative sidechain establishes aromatic interactions with H203^5.42^ and Y190^ECL2^ (not shown for the sake of clarity). Interestingly, NPA3^MOT^ is in close proximity to Y255^6.51^, which is located just above the highly conserved W252^6.48^ residue, which is commonly known as the toggle switch and directly implicated in GPCRs’ activation [7]. As described above, the presence of the 4-fluorobenzoyl chemical group at position 1 of motixafortide partially contributes to the less dynamic behavior of three bulky residues in the orthosteric binding site—Y116^3.32^, Y255^6.51^, and W252^6.48^—two of which have been experimentally characterized as important in CXCR4 activation (Y116^3.32^ and W252^6.48^) [14]. As mentioned above, mutagenesis studies at the CXCR4 receptor have shown that mutation of position Y116^3.32^ (Y116^3.32^S and Y116^3.32^A) decreases the receptor’s activation when stimulated with CXCL12 [12,13]. In the case of the toggle switch at TM6, even though the removal of the bulky residue—W252^6.48^A—does not significantly affect CXCL12 binding, it has a large effect on the chemokine ligand’s potency and efficacy [14]. Our simulations also identified the role of NPA3^MOT^ in restricting the conformations of these important residues associated with CXCR4 activation (Figure 6c). That is, the two synthetic chemical moieties of motixafortide work in tandem to restrict the conformations of important residues in the orthosteric binding site associated with CXCR4 activation (Figure 6).

## 3. Discussion

At present, the different diseases classified as cancer are considered to be a major public health problem worldwide and are responsible for the first or second highest numbers of deaths, depending on the region. Among the main types of cancer with the highest incidence are lung cancer, colorectal cancer, liver cancer, breast cancer, prostate cancer, and pancreatic cancer [15].

Due to the multifactorial nature of cancer, addressing most of its deleterious characteristics—including, mortality, morbidity, economic burden, and drug resistance—requires a multidisciplinary approach. Computational methods that allow the understanding of the functional mechanisms of biomolecules directly involved in cancer may offer detailed information (at a molecular level) that may be crucial to solving some of the aforementioned issues regarding cancer. Application of these types of computational methods, which have the ability to complement several experimental characterization techniques, has remained a relatively unexplored area in Mexico.

In this work, we applied several computational methods, including extensive all-atom molecular dynamics simulations, to characterize the binding mechanism of motixafortide—a promising compound currently involved in clinical trials for different types of cancer that regulates the CXCL12–CXCR4 signaling axis [16]. Motixafortide is a synthetic cyclic peptide that is currently involved in several preclinical studies related to therapies for several types of cancer, including breast and lung cancers, melanoma, and neuroblastoma [5,16]. Along these lines, recent therapeutic advances have demonstrated significant benefits of the administration of motixafortide, pembrolizumab (a programmed cell death protein 1 (PD-1) receptor blocker), and chemotherapy for the treatment of metastatic pancreatic ductal adenocarcinoma (PDAC) [5].

The primary pharmacological target of motixafortide is the CXC chemokine receptor type 4 (CXCR4)—a 352-residue class A G-protein-coupled receptor that is composed of seven transmembrane helices that span the lipid bilayer and that are connected by intra- and extracellular loops (Figure S1). CXCR4 is expressed in both hematopoietic and non-hematopoietic tissues, where it dictates relevant processes such as hematopoiesis, immune response, and regeneration, to name but a few [17]. Since CXCR4 plays a central role in tumor progression, angiogenesis, metastasis, and cell survival, its malfunction is directly associated with various forms of cancer, where generally it is not only overexpressed but also overactivated. Due to the physiological and pharmacological importance of the CXCL12–CXCR4 signaling axis, it has been extensively studied, and a clear view of the signal transduction determinants has emerged [11,18,19]. Extensive mutagenesis studies have proposed the involvement of different regions in the CXCR4 activation mechanism, as well as the residues that participate in the interaction with its endogenous ligand—the CXCL12 chemokine [11].

Herein, we investigate the interaction mechanism of motixafortide—a best-in-class antagonist of the CXCR4 receptor that has exhibited promising results in preclinical studies on pancreatic, breast, and lung cancers [5]. Using well-established computational techniques, we first investigated the conformational consequences of two ligands with opposite effects on the CXCR4 receptor—an antagonist (motixafortide) and an agonist (CXCL12). While the former stabilized inactive conformations of the receptor, the latter facilitated its activation; both complexes started from the same conformation of the CXCR4 receptor. Our extensive unbiased all-atom MD simulations indicated differential conformational changes triggered by the presence of the agonist and antagonist ligands, which are consistent with known information regarding agonist-bound and antagonist-bound systems. The changes included distinct conformations explored by the systems at particular structural motifs associated with the receptor’s activation, including the PIF, NPxxY, and toggle switch at TM6 motifs. Prompted by this adequate conformational characterization, we analyzed the interaction mechanism of motixafortide. We found that all six cationic residues (R1, R2, K7, K8, R11, and R14) were involved in charge–charge interactions with acidic residues in the orthosteric binding site of CXCR4, which may explain the outstanding ligand properties of motixafortide (high affinity and long ligand residence time; see Figure 4). Furthermore, our detailed analysis indicates that the two synthetic chemical moieties in motixafortide (the 4-fluorobenzoyl N-terminal capping group and the naphthalene derivative sidechain in position 3) play a central role in maintaining inactive conformations of important residues involved in CXCR4 activation. That is, these two aromatic bulky groups work in tandem to restrict the conformations of relevant CXCR4 signaling regions—particularly residues located at TM3 and TM6 (i.e., residues around the Y116^3.32^ and W252^6.48^ positions)—which is consistent with the proposed mechanism of CXCR4 activation. On the one hand, our simulations placed the 4-fluorobenzoyl group of motixafortide in the proximity of the aromatic residues W94^2.60^ and Y116^3.32^, where it mediates their adopted conformations (see Figure 6). Particularly, Y116^3.32^ has been found to play an important role in CXCR4 activation [12,13]. On the other hand, the naphthalene derivative group restricts the conformation of Y255^6.51^, which directly influences the behavior of the toggle switch at TM6 (W248^6.48^); changes in residue W^6.48^ have been suggested to facilitate GPCR activation [6,7]. These results further expand our knowledge about ways to control the central CXCL12–CXCR4 signaling axis by pinpointing specific interacting loci at the orthosteric site of CXCR4. Notably, the citrulline residues do not seem to play an important role in mediating the interaction between motixafortide and the CXCR4 receptor.

Our results not only provide molecular-level information about the interaction mechanism by which motixafortide inhibits the CXCL12–CXCR4 signaling axis, but also provide essential information to rationally design CXCR4 inhibitors that retain the outstanding pharmacological features of motixafortide.

## 4. Materials and Methods

### 4.1. Ballesteros–Weinstein Numbering for Class A GPCRs

Herein, Ballesteros–Weinstein numbering is used as a superscript to label the different positions of the TM helices [20,21]. This nomenclature is utilized in class A GPCRs and uses the highly conserved residues at each of seven transmembrane helices. In this scheme, the first number represents the number of the TM helix (1–7), while the second indicates the residue’s position relative to the most conserved position, which is denoted by the number 50; in general, the highly conserved residues for each transmembrane are N^1.50^, D^2.50^, R^3.50^, W^4.50^, P^5.50^, P^6.50^, and P^7.50^.

### 4.2. Construction of the CXCL12/CXCR4 Complex

Structural information regarding the CXCL12/CXCR4 complex has not yet been determined experimentally. To obtain an initial conformation of the CXCL12/CXCR4 protein system, the structure deposited in the PDB database with the access code 4RWS was used [19]. This X-ray structure contains the human CXCR4 bound to a viral chemokine called vMIP-II (a CXCR4 antagonist). Moreover, the NMR structure of the CXCL12 ligand was obtained from the same database with the access code 1SDF.pdb [22]. The initial molecular pose of CXCL12 in the orthosteric ligand binding site of CXCR4 was obtained by a structural alignment of the two chemokines (Figure S8; see also the detailed description in the Supplementary Materials).

### 4.3. Construction of the Motixafortide/CXCR4 Complex

The structural information of motixafortide (BL-8040) has not been determined; therefore, to generate an initial structure of the motixafortide/CXCR4 complex, we used the crystallographic information of the complex formed by the cyclic peptide antagonist CVX15 and the human CXCR4 (3OE0 PDB identification code) [18]. The initial tertiary structure of motixafortide was constructed by mapping the respective sidechains on the backbone structure of the CVX15 antagonist (see Figure S8). Lastly, the synthetic and non-essential amino acids of motixafortide were constructed using VMD [23,24].

### 4.4. Preparation of the Protein Complexes

The protein complexes investigated—CXCL12/CXCR4 and motixafortide/CXCR4— were embedded in a hydrated (TIP3 water model) lipid bilayer of 1-palmitoyl-2-oleoyl-sn-glycero-3-phosphocholine (POPC). Both complexes were then ionized using a 0.15 M salt concentration of NaCl. The biomolecular systems were prepared using the VMD software [23,24]. The size of the resulting systems was ~90,000 atoms (Figure S2; see also the detailed description in the Supplementary Materials).

### 4.5. All-Atom Molecular Dynamics Simulations

Molecular dynamics (MD) simulations of the two complex systems were conducted for 1.0 μs, and the software used was NAMD with the all-atom CHARMM36 and CGenFF force fields [25,26]. The conditions used to simulate a physiological environment in the systems were 310.15 K and 1 atm of pressure. Full electrostatics were evaluated using PME techniques with grid spacing < 1.0 Å in each dimension and a fourth-order interpolation. Bond lengths involving hydrogen atoms were constrained to their equilibrium values using the SHAKE algorithm. All unbiased MD simulations were performed with a 2.0 fs time step [27]. Detailed descriptions of the computational simulations carried out here have been previously published by our group [27,28,29] (see also the detailed descriptions in the Supplementary Materials [30,31,32,33,34,35,36]).

## 5. Conclusions

Using extensive unbiased molecular dynamics, we elucidated the mechanism by which motixafortide interacts with the CXCR4 receptor and stabilizes inactive states of the receptor, at the molecular level. Our results indicate the crucial role of the cationic residues as well as the synthetic chemical moieties of motixafortide.

## Data Availability

Not applicable.

## Supplementary Materials

The following supporting information can be downloaded at: https://www.mdpi.com/article/10.3390/ijms24054393/s1.
