# Supplementary material for: Structural Basis of the Binding Mode of the Antineoplastic Compound Motixafortide (BL-8040) in the CXCR4 Chemokine Receptor
Source: Int J Mol Sci. 2023 Feb 23;24(5):4393. doi: 10.3390/ijms24054393 (PMC10001991; doi:10.3390/ijms24054393)
Supplement: Supplementary file 1 [file ijms-24-04393-s001.zip › ijms-2162060-supplementary.pdf]

International Journal of *Molecular Sciences*

MDPI

Supplementary Material

Structural Basis of the Binding Mode of the Antineoplastic Compound Motixafortide (BL-8040) in the CXCR4 Chemokine Receptor

Mariana Rebolledo-Bustillo [1,†], David Garcia-Gomez [1,†], Eliud Morales Dávila [1], María Eugenia Castro [2], Norma A. Caballero [3], Francisco J. Melendez [1], Victor M. Baizabal-Aguirre [4], Brenda L. Sanchez-Gaytan [2] and Jose Manuel Perez-Aguilar [1,*]

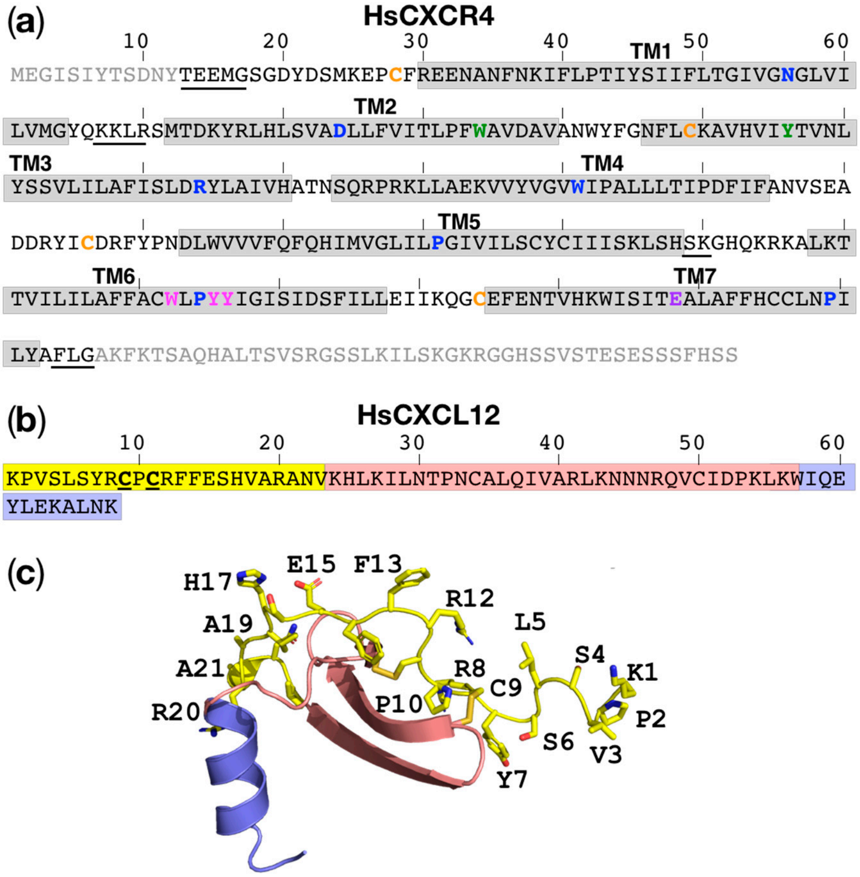

Figure S1. General information regarding the investigated systems. (**a**) Primary sequence of the human CXCR4 receptor where the seven TM helices are indicated by grey rectangles. The most conserved residue at each TM helix is colored blue. The segment of CXCR4 not considered in the tertiary structure investigated here is colored grey. The residues added at different regions of the CXCR4 structure are underlined. (**b**) Primary structure of the CXCL12 ligand where the N-terminal segment, β-sheet region, and C-terminal helix are colored yellow, salmon, and violet, respectively. (**c**) The tertiary structure of CXCL12 using the same color code is depicted (1SDF.pdb). Residues at the N-terminal segment are indicated.

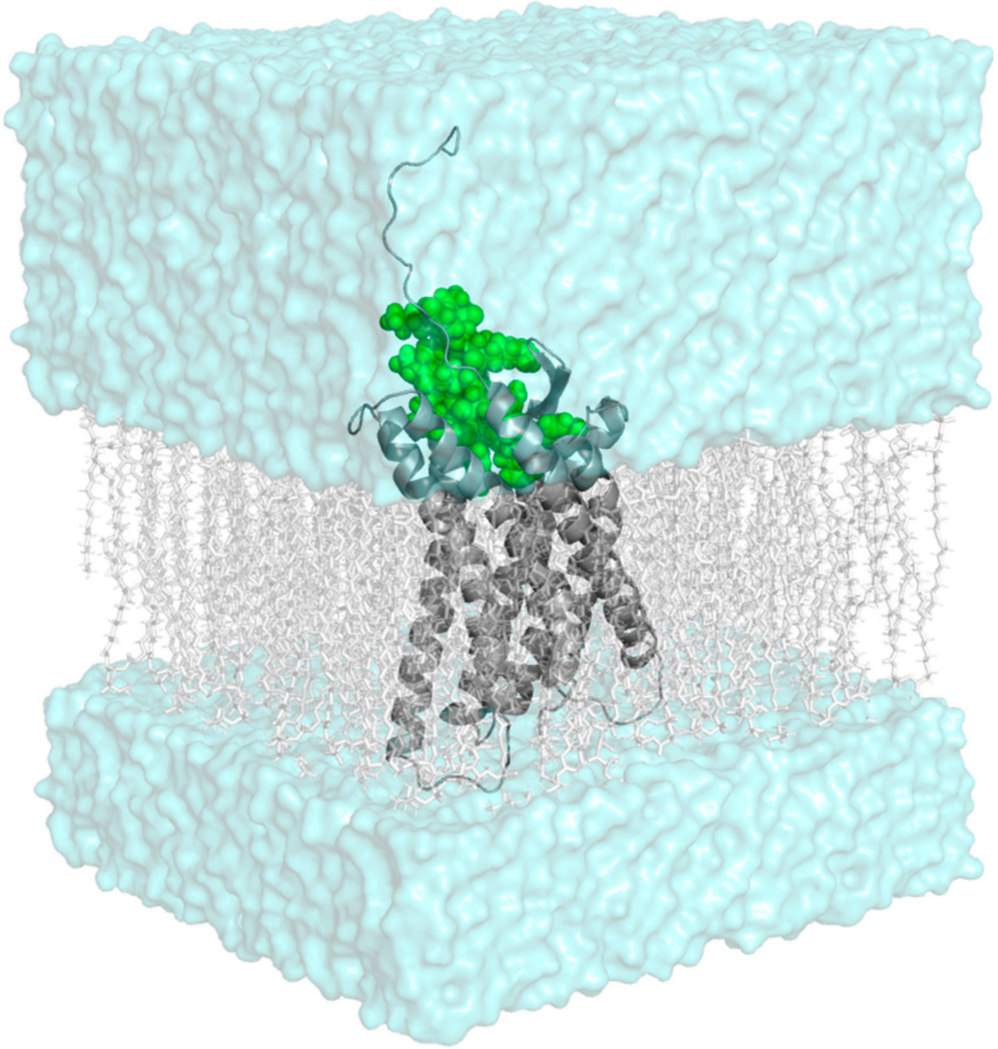

Figure S2. Representation of the system motixafortide/CXCR4 investigated by all-atom MD simulations. An example of the initial structure one of the systems investigated in this work. The structure of the CXCR4 receptor is represented as grey ribbons and the structure of the motixafortide ligand is depicted as green spheres. Water is shown as a surface representation while the POPC molecules that constitute the lipid bilayer are depicted as white sticks. The size of the system is ~90,000 atoms. .

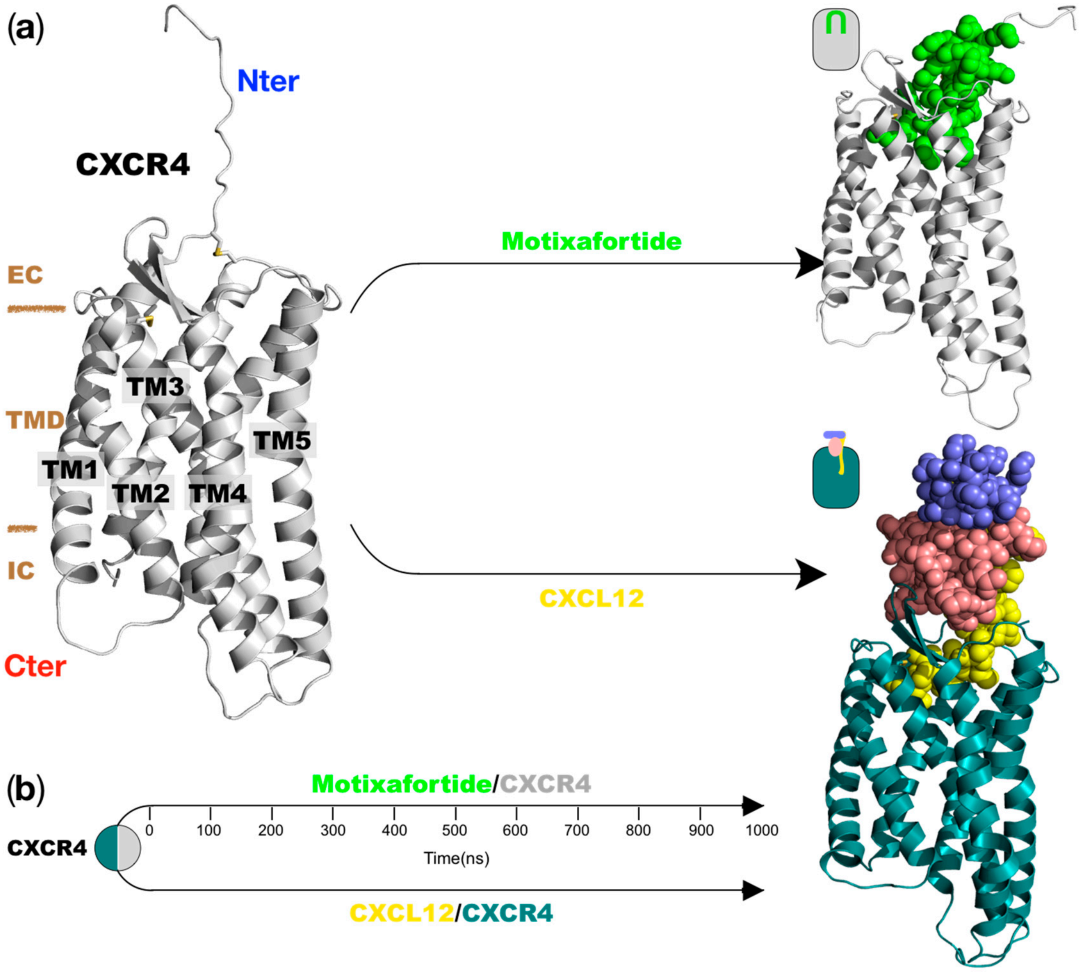

Figure S3. Atomistic MD simulations were utilized to characterize the CXCR4 systems. (**a**) Starting from the same CXCR4 structure, complexes with motixafortide (antagonist) and CXCL12 (agonist were generated. In the motixafortide/CXCR4 protein complex, the ligand is colored green while the receptor is colored light gray. In the case of the CXCL12/CXCR4 complex, the receptor is colored teal while the ligand is colored using the same color code as in Figure S1. (**b**) 1000ns-long unbiased MD simulations were carried out for each CXCR4 complex.

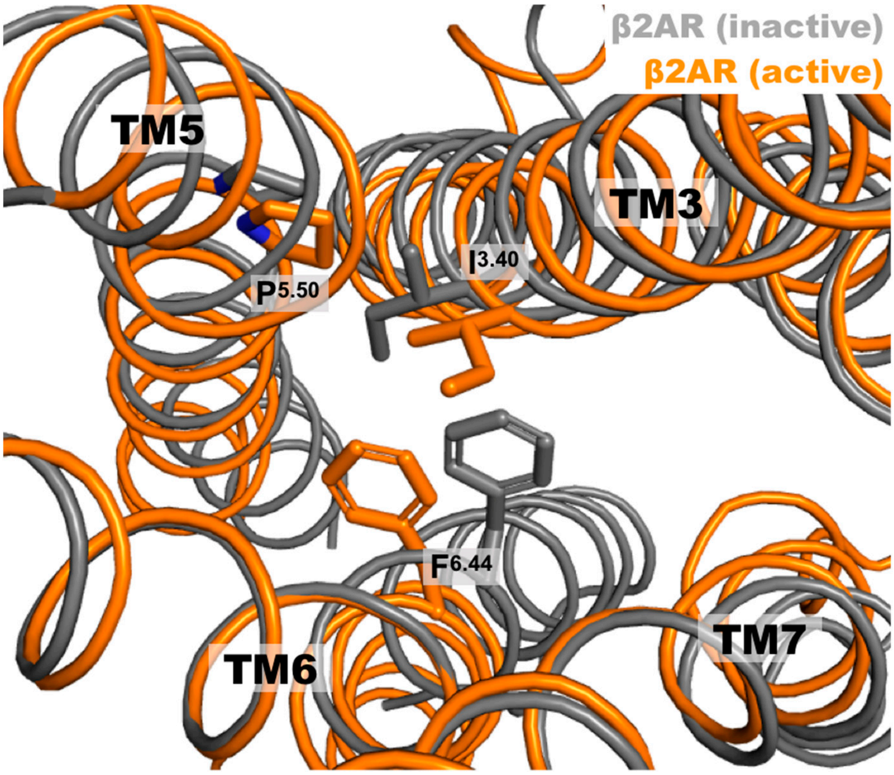

Figure S4. Changes in the PIF motif. Comparison of the conserved PIF motif ($P^{5.50}$ $I^{3.40}$ $F^{6.44}$) in a prototypical class A GPCR, the β2 adrenergic receptor (β2AR). Relative to the inactive receptor's conformation, position $F^{6.44}$, moves toward TM5. The inactive β2AR structure is taken from the 2RH1.pdb file while that of the active conformation was taken from 4LDE.pdb.

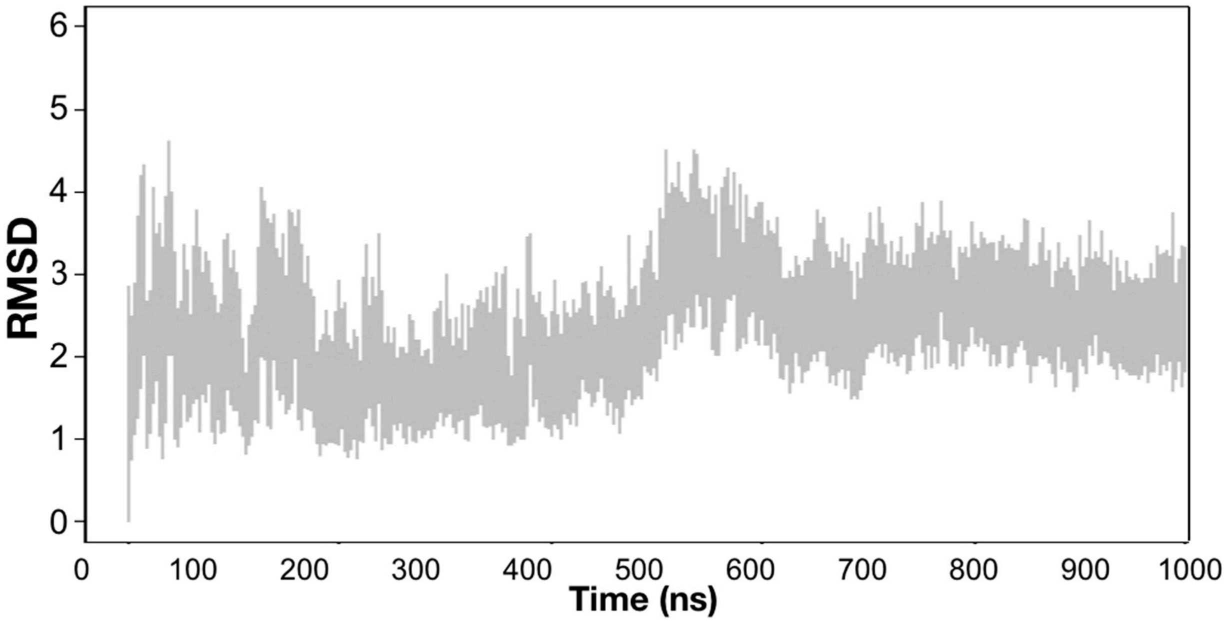

Figure S5. RMSD calculation. Calculation of the RMSD values for the backbone atom of motixafortide. Time evolution plot of the RMSD values for the backbone atoms of the MT helices of the CXCR4 receptor.

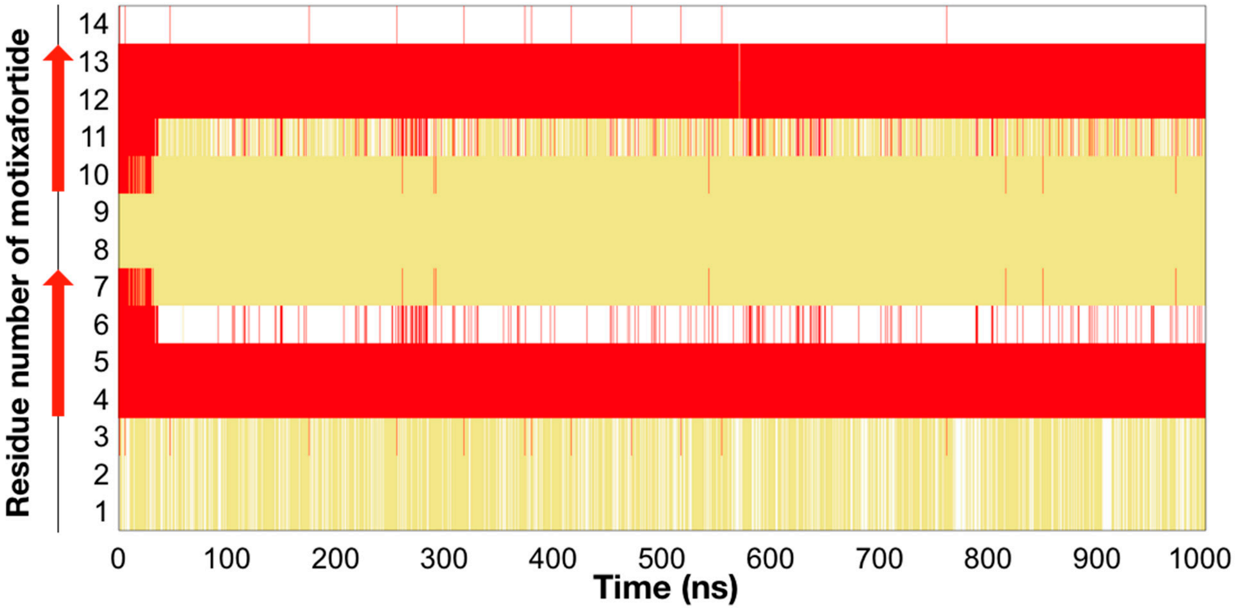

Figure S6. Time evolution of the secondary structure content of motixafortide. The time evolution of the secondary structure content for the 14-residue peptide motixafortide along the 1.0 μs simulation as calculated in VMD using the program STRIDE. The color code for the different secondary structure type is as follows: β sheet in red, extended configuration or isolated bridge in khaki, and random coil in white. The secondary structure of the original structure is depicted next to the residue number.

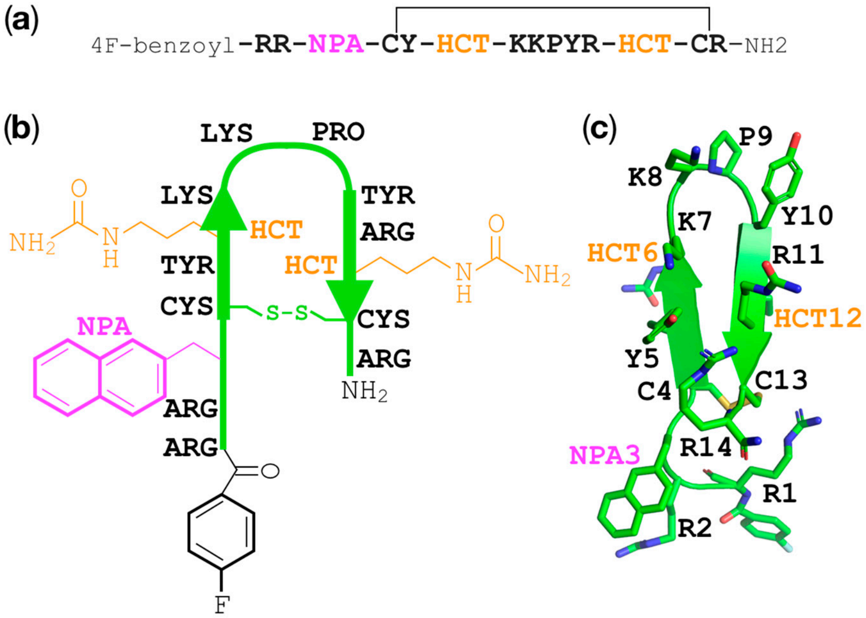

Figure S7. Structure of motixafortide. (**a**) The residue sequence of motixafortide is shown where HCT is the amino-acide citrulline. The disulfide bond present in motixafortide is also indicated. (**b**) Scheme of the structure of motixafortide where the non-standard α-amino-acids as well as the N-terminal capping group are depicted. (**c**). The initial structure of motixafortide is shown.

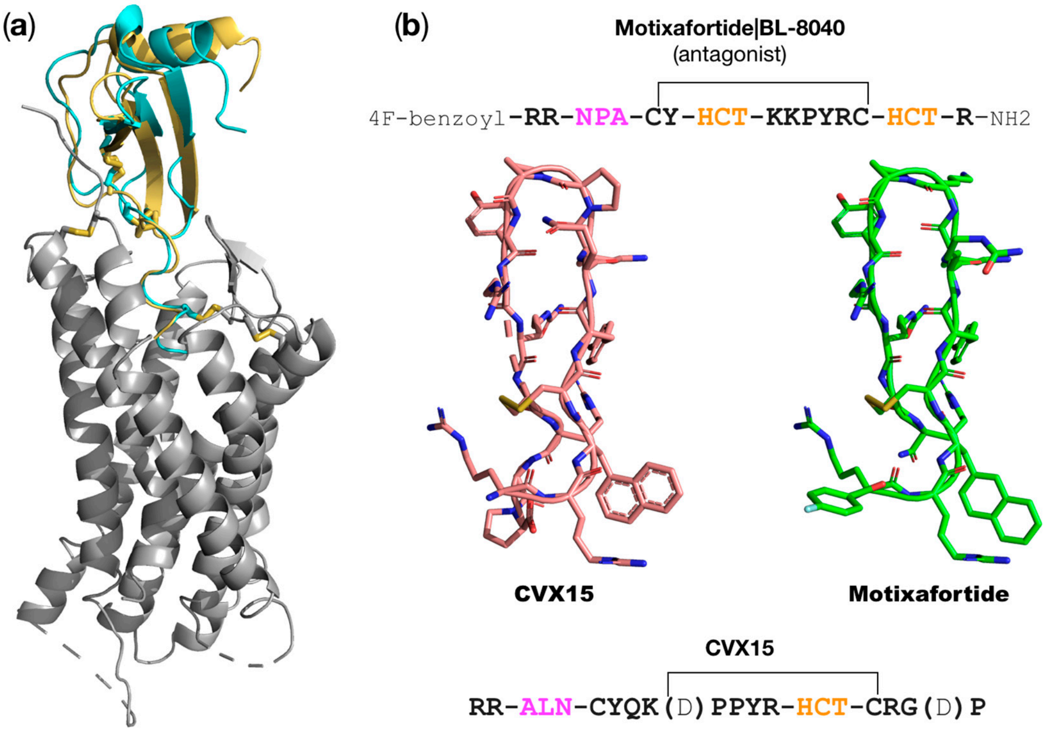

Figure S8. Experimental information utilized to generate the initial structure of the CXCR4 protein complexes. (a) To generate the initial molecular pose of the cxcl12/CXCR4, the structure of the cxcl12 from the SDF1.pdb (yellow) was structurally aligned to the complex structure of the XXX/CXCR4 from the XXX.pdb. (b) The structure of the motixafortide was constructed using the structural information of the CVX15/CXCR4 from the **CCCC**.pdb.

Construction of the CXCR4 protein complexes

The structure of the human CXCR4 receptor was taken from the structure deposited in the PDB database with the 4RWS accession code. Residues located at the N-terminal, T13 to G22, were included as an unstructured extended segment—using the program PyMOL [1] —to include residues proposed to play an important role in the interaction of CXCR4 with the endogenous ligand CXCL12. The residues added at different regions of the CXCR4 sequence are underlined in the sequence of the receptor in the revised figure S1. The missing segments were added using the loop modeling capabilities of MODELLER [2] and are, K67 to R70 (ICL1), S229 to K230 (ICL3) and F304 to G306 (C-terminus). The resulting final structure comprises the segment T13 to G306. The final structure was oriented according to the information from the CXCR4 receptor in the OPM database [3].

Construction of the CXCL12/CXCR4 Complex. Structural information regarding the CXCL12/CXCR4 complex has not yet been determined experimentally. To obtain an initial conformation of the CXCL12/CXCR4 protein system, the structure deposited in the PDB database with the access code 4RWS was used [4]. This X-ray structure contains the human CXCR4 bound to a viral chemokine called vMIP-II (CXCR4 antagonist). Also, the NMR structure of CXCL12 ligand was obtained from the same database with the access code 1SDF.pdb [5]. The initial molecular pose of CXCL12 in the orthosteric ligand binding site of CXCR4 was obtained by a structural alignment of the two chemokines (Figure S8). The CXCL12/CXCR4 complex structure was subject to a preparation protocol describe below.

Construction of the motixafortide/CXCR4 Complex. The structural information of motixafortide (BL-8040) has not been determined, therefore, to generate an initial

structure of the motixafortide/CXCR4 complex, we used the crystallographic information of the complex formed by the cyclic peptide antagonist CVX15 and the human CXCR4 (3OE0 PDB identification code) [6]. The initial tertiary structure of motixafortide was constructed by mapping the respective sidechains on the backbone structure of the CVX15 antagonist, see Figure S8. Lastly, the synthetic and non-essential amino acids of motixafortide were constructed using VMD [7]. The motixafortide/CXCR4 complex structure was subject to a preparation protocol describe below.

Preparation of the protein complexes. The protonation states of the amino acid residues of CXCR4 and the ligands were chosen as those more likely to be present at pH = 7.0. The protein complexes investigated—CXCL12/CXCR4 and motixafortide/CXCR4— were embedded in a hydrated (TIP3 water model) lipid bilayer of 1-palmitoyl-2-oleoyl-sn-glycero-3-phosphocholine (POPC). The POPC lipid bilayer was constructed using the Membrane Plugin in the VMD program [7]. The position of the CXCR4 complexes with respect to the lipid bilayer was selected according to the suggested limits from the OPM database. Lipids overlapping with the protein complexes were removed and ions were added to obtain neutral systems as well as to replicate a 0.15 M salt concentration using VMD. In the case of the CXCL12/CXCR4 system 55 sodium ions and 65 chloride ions were added while in the case of the motixafortide/CXCR4 system 55 sodium ions and 62 chloride ions were included. The systems were prepared using the VMD program [7].

Protocol for the preparation of the systems before the atomistic unbiased MD simulations

Six preliminary phases were carried out for the preparation of the complex systems (using NAMD) as follows:

Step 1. The systems were minimized by 10,000 steps to avoid possible clashes using the conjugate gradient algorithm. The minimization was followed by 100,000 MD steps with a timestep of 0.5 fs in the NVT ensemble. Here, the positions of the atoms in the systems were kept fixed except for the lipids tails and protein backbone atoms.

Step 2. Minimization of the system by 20,000 steps with a timestep of 0.5 fs is performed. The minimization was followed by 250,000 MD steps where protein atoms were fixed and strong restraints (harmonic potential with a force constant of 1.0 kcal/mol A^2) in the lipid atoms fixed in the previous step are applied (NPT ensemble). Also, external forces are applied to the water molecules to prevent them for entering the lipid membrane.

Step 3. 250,000 MD steps with strong restraints (harmonic potential with a force constant of 1.0 kcal/mol A^2) in the protein heavy atoms using a timestep of 1.0 fs are carried out. External forces are applied to the water molecules to prevent them for entering the lipid membrane.

Step 4. 250,000 MD steps with strong restraints (harmonic potential with a force constant of 1.0 kcal/mol A^2) in the protein heavy atoms using a timestep of 2.0 fs in the NPT ensemble are carried out.

Step 5. 250,000 MD steps with medium restraints (harmonic potential with a force constant of 0.5 kcal/mol A^2) in the protein heavy atoms using a timestep of 2.0 fs in the NPT ensemble are carried out.

Step 6. 250,000 MD steps with mild restraints (harmonic potential with a force constant of 0.1 kcal/mol A^2) in the protein heavy atoms using a timestep of 2.0 fs in the NPT ensemble are carried out.

All-atom molecular dynamics simulation.

The unbiased MD simulations were performed with the all-atom CHARMM36 force field with CMAP corrections for proteins and lipids [8] using NAMD [9]. The production phase (1000 ns) was carried out using the NPT ensemble and Langevin dynamics and the hybrid Nosé–Hoover Langevin piston were used to maintain constant temperature (37 °C) and constant pressure (1 atm), respectively [10]. The particle-mesh Ewald [11] technique

was utilized to evaluate the electrostatic interactions with grid spacing <1.0 Å in each dimension and a fourth-order interpolation. The periodic boundary conditions were applied in 3 orthogonal dimensions. Bond lengths involving hydrogen atoms were constrained to their equilibrium values using the SHAKE algorithm [12]. The MD simulations were performed with a 2.0 fs time step. The systems were simulated for 500,000,000 steps with a step size of 2.0 fs, to obtain a total trajectory of 1,000 ns (1.0 μs).

References

1. Schrödinger, L.; DeLano, W. PyMOL. Available online: https://www.pymol.org/pymol.
2. Fiser, A.; Šali, A. Modeller: Generation and Refinement of Homology-Based Protein Structure Models. **2003**, 461–491. https://doi:10.1016/S0076-6879(03)74020-8.
3. Lomize, M.A.; Lomize, A.L.; Pogozheva, I.D.; Mosberg, H.I. OPM: Orientations of Proteins in Membranes Database. *Bioinformatics* **2006**, *22*, 623–625. https://doi:10.1093/bioinformatics/btk023.
4. Qin, L.; Kufareva, I.; Holden, L.G.; Wang, C.; Zheng, Y.; Zhao, C.; Fenalti, G.; Wu, H.; Han, G.W.; Cherezov, V.; et al. Crystal Structure of the Chemokine Receptor CXCR4 in Complex with a Viral Chemokine. *Science* **2015**, *347*, 1117–1122. https://doi.org/10.1126/science.1261064.
5. Crump, M.P.; Gong, J.H.; Loetscher, P.; Rajarathnam, K.; Amara, A.; Arenzana-Seisdedos, F.; Virelizier, J.L.; Baggiolini, M.; Sykes, B.D.; Clark-Lewis, I. Solution Structure and Basis for Functional Activity of Stromal Cell-Derived Factor-1; Dissociation of CXCR4 Activation from Binding and Inhibition of HIV-1. *EMBO J.* **1997**, *16*, 6996–7007. https://doi.org/10.1093/emboj/16.23.6996.
6. Wu, B.; Chien, E.Y.T.; Mol, C.D.; Fenalti, G.; Liu, W.; Katritch, V.; Abagyan, R.; Brooun, A.; Wells, P.; Bi, F.C.; et al. Structures of the CXCR4 Chemokine GPCR with Small-Molecule and Cyclic Peptide Antagonists. *Science* **2010**, *330*, 1066–1071. https://doi.org/10.1126/science.1194396.
7. Humphrey, W.; Dalke, A.; Schulten, K. VMD: Visual Molecular Dynamics. *J. Mol. Graph.* **1996**, *14*, 33–38. https://doi:10.1016/0263-7855(96)00018-5.
8. Mackerell, A.D.; Feig, M.; Brooks, C.L. Extending the Treatment of Backbone Energetics in Protein Force Fields: Limitations of Gas-Phase Quantum Mechanics in Reproducing Protein Conformational Distributions in Molecular Dynamics Simulations. *J. Comput. Chem.* **2004**, *25*, 1400–1415. https://doi:10.1002/jcc.20065.
9. Nelson, M.T.; Humphrey, W.; Gursoy, A.; Dalke, A.; Kalé, L.V.; Skeel, R.D.; Schulten, K. NAMD: A Parallel, Object-Oriented Molecular Dynamics Program. *Int. J. Supercomput. Appl. High Perform. Comput.* **1996**, *10*, 251–268. https://doi.org/10.1177/109434209601000401.
10. Feller, S.E.; Zhang, Y.; Pastor, R.W.; Brooks, B.R. Constant Pressure Molecular Dynamics Simulation: The Langevin Piston Method. *J. Chem. Phys.* **1995**, *103*, 4613–4621. https://doi:10.1063/1.470648.
11. Darden, T.; York, D.; Pedersen, L. Particle Mesh Ewald: An N ·log(N) Method for Ewald Sums in Large Systems. *J. Chem. Phys.* **1993**, *98*, 10089–10092. https://doi:10.1063/1.464397.
12. Ryckaert, J.P.; Ciccotti, G.; Berendsen, H. J. C. Numerical integration of the cartesian equations of motion of a system with constraints: molecular dynamics of *n*-alkanes. *Journal of Computational Physics* **1977**, *23 (3), 327-41*. https://doi.org/10.1016/0021-9991(77)90098-5.
